# Antigen-Specific Urinary Immunoglobulin in Reservoir Hosts of Leptospirosis

**DOI:** 10.3390/vetsci8090178

**Published:** 2021-09-01

**Authors:** Jarlath E. Nally, Richard L. Hornsby, David P. Alt

**Affiliations:** Infectious Bacterial Diseases Research Unit, National Animal Disease Center, Agricultural Research Service, United States Department of Agriculture, Ames, IA 50010, USA; richard.hornsby@usda.gov (R.L.H.); david.alt@usda.gov (D.P.A.)

**Keywords:** *Leptospira*, leptospirosis, urinary immunoglobulin, reservoir hosts

## Abstract

Domestic and wildlife animal species act as reservoir hosts of leptospirosis, a global zoonotic disease affecting more than 1 million people annually and causing significant morbidity and mortality in domestic animals. In contrast to incidental hosts which present with an array of clinical manifestations, reservoir hosts are typically asymptomatic and can shed leptospires from chronically infected kidneys via urine for extended periods of time. Renal excretion of leptospires occurs despite evidence of a humoral and cellular immune response and is reflective of the unique biological equilibrium that exists between certain animal species and specific serovars of *Leptospira*. Here, we demonstrate that urinary excretion of leptospires is accompanied by the presence of antigen-specific urinary immunoglobulin. In rats experimentally infected with *L*. *interrogans* serovar Copenhageni using the intraperitoneal or conjunctival route of inoculation, urinary immunoglobulin (Ig) G specific for protein antigens was detectable within 1 week. Rat urinary IgG was not bound to urinary-derived leptospires. In cattle that were naturally exposed to, and infected with, *L*. *borgpetersenii* serovar Hardjo, urinary IgA specific for protein antigens was detected. Collectively, these results demonstrate that urinary excretion of immunoglobulin specific for leptospires is a hallmark of reservoir hosts of infection.

## 1. Introduction

Pathogenic leptospires of the genus *Leptospira* are the causative agent of leptospirosis, a global zoonotic disease infecting more than 1 million people annually [1]. Disease transmission is maintained by domestic and wildlife animal species which act as reservoir hosts of infection and excrete leptospires colonizing the renal tubules via urine into the environment where they can persist in suitable moist conditions [2]. Incidental hosts can be infected by direct contact with urine from reservoir hosts or indirectly by contact with contaminated water or other environmental sources since leptospires can actively penetrate mucosal surfaces or breaches of the skin. Though reservoir hosts are generally asymptomatic, animal leptospirosis in domestic animal species can also result in significant morbidity and mortality; bovine leptospirosis causes reproductive failure, abortion, stillbirth, and loss of milk production [3]. Serologic reactivity detected using the microscopic agglutination test (MAT) is most often used to establish exposure of an animal to pathogenic leptospires, but this is of limited value in identifying reservoir hosts of infection since active shedding of leptospires can occur in the absence of a detectable MAT titer [4,5].

A unique biological equilibrium exists between certain animal reservoir hosts and specific serovars of *Leptospira*, and, as exemplified by *L*. *interrogans*, serovar Copenhageni in rats [6,7]. In contrast with experimentally infected guinea pigs which suffer an acute lethal disease, experimentally infected rats remain clinically asymptomatic and can become persistent carriers of leptospires despite evidence of a humoral and cellular immune response [8,9,10]. It is well established that pathogenic leptospires regulate gene, protein, and protein post-translation modifications in response to a range of environmental cues that reflect those encountered during host infection, and it is hypothesized that such regulatory events modulate the host response to infection [11,12,13,14,15,16]. Evaluation and characterization of urine from experimentally infected rats and naturally infected sea-lions confirm changes in the urinary proteome and demonstrate the presence of host-derived proteomic biomarkers of infection [17,18,19].

Previous work has demonstrated that genes encoding immunoglobulin are increased in expression in kidneys derived from experimentally infected rats compared to non-infected controls [15]. In this work, we demonstrate the presence of urinary immunoglobulin, not only in experimentally infected rats, but naturally infected cattle. In both cases, urinary immunoglobulin is specific for protein antigens of *Leptospira*.

## 2. Materials and Methods

### 2.1. In Vitro Cultivated Bacteria

*L*. *interrogans* serovar Copenhageni strain RJ19115 was cultivated under standard conditions at 29 °C in EMJH medium (BD Difco, Sparks, MD, USA). *L*. *borgpetersenii* serovar Hardjo strain JB197 was cultivated under standard conditions at 29 °C in T80/40/LH medium prepared as previously described with the following modifications: 5-FU was used at 100 μg/mL and Nalidixic acid was not used [20].

### 2.2. Experimental Infection of Rats and Urine Collection

All animal experimentation was conducted in accordance with protocols as reviewed and approved by the Animal Care & Use Committee at the National Animal Disease Center, and as approved by USDA Institutional guidelines. Outbred male Sprague–Dawley rats (*n* = 12) (Envigo, Indianapolis, IN, USA) or inbred female Fisher 344 rats (*n* = 6) (Strain F344/NHsd, Envigo, Indianapolis, IN, USA), of approximately 4–5 weeks of age, were experimentally infected with 1 × 10^7^ low-passage *L*. *interrogans* strain RJ19115 by intraperitoneal injection in a final volume of 0.5 mL. Non-infected control rats (*n* = 10) received 0.5 mL of culture medium. Alternatively, rats were anesthetized with isoflurane and 25 µL of media containing 2.5 × 10^6^ leptospires were administered to each nasal cavity and the surface of each eye, for three consecutive days (*n* = 6) [21,22,23]. In order to collect urine samples, rats were housed individually in a metabolism cage for approximately 1 h immediately after receiving furosemide.

### 2.3. Bovine Samples

Bovine sera and urine samples were collected and processed for MAT and the fluorescent antibody test (FAT), as previously described [5]. Bovine urine was stored at −20 °C after processing for FAT and prior to electrophoresis and immunoblotting.

### 2.4. Protein Electrophoresis and Immunoblotting

Urine samples were processed for one-dimensional (1-D) SDS-PAGE on 12% acrylamide gels (BioRad, Hercules, CA, USA), as per the manufacturer’s guidelines. Proteins were visualized by staining with Sypro Ruby (Invitrogen, Carlsbad, CA, USA). For immunoblotting, samples were transferred to the Immobilon-P transfer membrane (Millipore, Bedford, MA, USA). For rat urine samples, membranes were blocked overnight at 4 °C with 5% non-fat dried milk in phosphate-buffered saline (PBS) containing 0.1% Tween 20 (PBS-T). Membranes were then incubated with horseradish-peroxidase anti-rat IgG for 1 h at room temperature (diluted 1 in 5000 with PBS-T). For bovine urine samples, membranes were blocked overnight at 4 °C with StartingBlock (PBS) blocking buffer (ThermoFisher, Carlsbad, CA, USA). Membranes were then incubated with horseradish-peroxidase anti-bovine IgG (Bethyl, Montgomery, TX, USA) or anti-bovine IgA (Bethyl, Montgomery, TX, USA) diluted 1 in 5000 or 2500, respectively, in blocking buffer, for 1 h at room temperature (RT). After washing, bound conjugates were detected using Clarity Western ECL substrate (BioRad, Hercules, CA, USA) and images were acquired using a Bio-Rad ChemiDoc MP imaging system (BioRad, Hercules, CA, USA).

For detection of urinary immunoglobulin specific antigens in rats, strain RJ19115 was cultured to the mid–late log phase (~1 × 10^8^ leptospires/mL), harvested by centrifugation (10,000× *g*, 4 °C, 30 min) and washed twice with PBS. Immunoblotting was performed with approximately 1 × 10^7^ leptospires per lane which was blocked overnight at 4 °C with 5% non-fat dried milk in PBS-T. Membranes were then probed directly with neat urine collected from individual rats at indicated time points, or sera diluted at 1 in 1000 in PBS-T. Reactive antigens were detected with horseradish peroxidase (HRP) anti-rat IgG (1 in 5000 in PBS-T), as described above. For detection of urinary immunoglobulin specific antigens in cattle, strain JB197 was cultured to the mid–late log phase (~1 × 10^8^ leptospires/mL), harvested by centrifugation (10,000× *g*, 4 °C, 30 min) and washed twice with PBS. Immunoblotting was performed with approximately 1 × 10^7^ leptospires per lane which was blocked for 30 min with StartingBlock (PBS) Blocking buffer at room temperature before being probed directly with neat urine overnight at 4 °C. Reactive antigens were detected with HRP anti-bovine IgA (1 in 25,000 in PBS-T), as described above.

### 2.5. Fluorescent Antibody Test (FAT)

A 10-µL aliquot of rat urine was placed on a glass slide with a 7-mm well, performed in duplicate. Urine was allowed to air dry in the dark and fixed in acetone for 15 min at RT. Slides were placed in a humid chamber and 20 µL of rat serum (post-infection week 8) or 20 µL of urine (post-infection week 4 and post-infection week 8) from a chronically infected rat, diluted 1 in 100 in PBS, was added to each well. Slides were incubated at 37 °C for 1 h. Slides were washed for 10 min in PBS at RT with gentle rocking. Slides were dried and 50 µL of donkey α-rat IgG-Alexa 594 (Invitrogen, Carlsbad, CA, USA) at 1 in 1000 dilution, and 10 µL of 4’,6-diamidino-2-phenylindole (DAPI) (1 in 500 in PBS) was applied to each well. Slides were incubated at 37 °C for 1 h. Slides were washed for 10 min in PBS at RT with gentle rocking. To determine if urinary-derived antibodies were bound to urinary-derived leptospires, slides were also tested directly with donkey α-rat IgG-Alexa 594 (Invitrogen, Carlsbad, CA, USA). Slides were also tested with serum and urine from non-infected control rats. Slides were dried and coverslip mounted with 20 µL of ProLong Gold antifade reagent (Invitrogen, Carlsbad, CA, USA), diluted 1 in 3 in PBS, which allowed them to cure overnight in the dark. Staining was evaluated at 400X with a BioTek^®^ Cytation 5 imaging reader (Winooski, VT, USA) using Fluorescein isothiocyanate (FITC) (excitation, 450−490 nm, emission, 520 nm), Texas Red (excitation, 560 nm, emission, 600 nm), and DAPI (excitation, 356nm, emission, 409 nm) fluorescent filters.

## 3. Results

**Figure 1:**
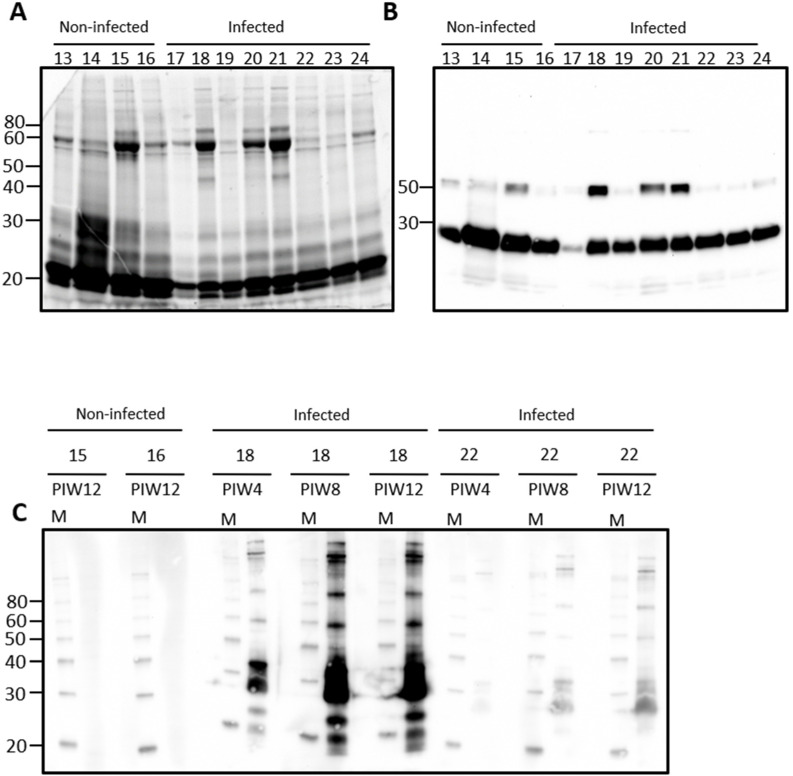
Representative total protein content (**A**) or IgG detection (**B**) in rat urine. Urine was collected from outbred male rats at post-infection week (PIW) four after inoculation by the intraperitoneal route. Urinary IgG specific for leptospires was demonstrated in infected rats by immunoblotting of leptospires (**C**) with urine collected at PIW 4, 8, and 12 in infected rats (numbered 17–24) compared to non-infected controls (numbered 13–16). Molecular mass markers (M) are indicated in kDa.

Urine from rats contains a large number of proteins, Figure 1A. Immunoblotting of urine samples from rats experimentally infected with leptospires, and non-infected controls, demonstrates that IgG is also readily detected in urine samples, Figure 1B. Attempts to identify urinary IgA were not satisfactory using antibodies specific for rat-IgA (data not shown). Significant variability was observed for the detection of IgG heavy and light chains and, therefore, representative samples were chosen to determine the specificity of urinary IgG for antigens of *Leptospira*, Figure 1C. In rat 18, for which both IgG heavy and light chain was readily detected by immunoblot in Figure 1B, urinary IgG specific for leptospires was present by post-infection week (PIW) 4 and increased at PIW8 and PIW12. In rat 22, for which IgG light chain was readily detected but not heavy chain, much less reactivity specific for leptospires was detected at PIW4, though increased reactivity was also apparent at PIW8 and PIW12. No specific reactivity for leptospires was detected in urinary IgG from non-infected control rats which contained IgG heavy and light chains.

**Figure 2:**
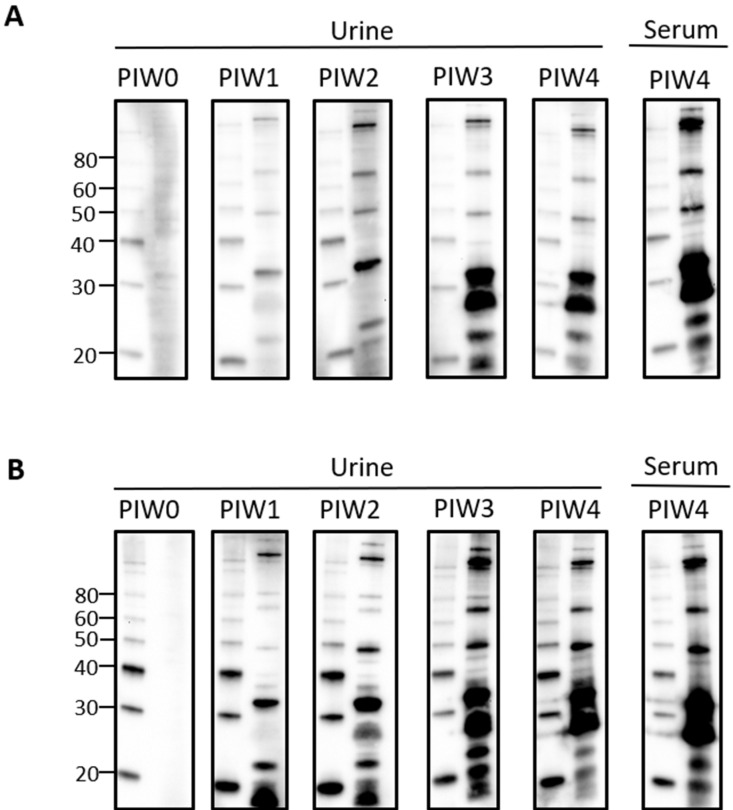
Time course for the detection of urinary IgG specific for leptospires in intraperitoneally infected rats. Representative immunoblots of leptospires with urine collected from (**A**) rat 18 or (**B**) rat 21 at PIW 0, 1, 2, 3, and 4, or serum collected at PIW4. Molecular mass markers (M) are indicated in kDa and are shown in the left lane of each immunoblot compared to the antigen of leptospires in the right lane.

Given that urinary IgG specific for leptospires was clearly detected by PIW4, a time course was performed with urine collected from intraperitoneally infected rats at weekly time points from PIW0 to PIW4. Representative immunoblots showed that urinary IgG was detected as early as PIW1, and increased over time, as seen in Figure 2A,B.

**Figure 3:**
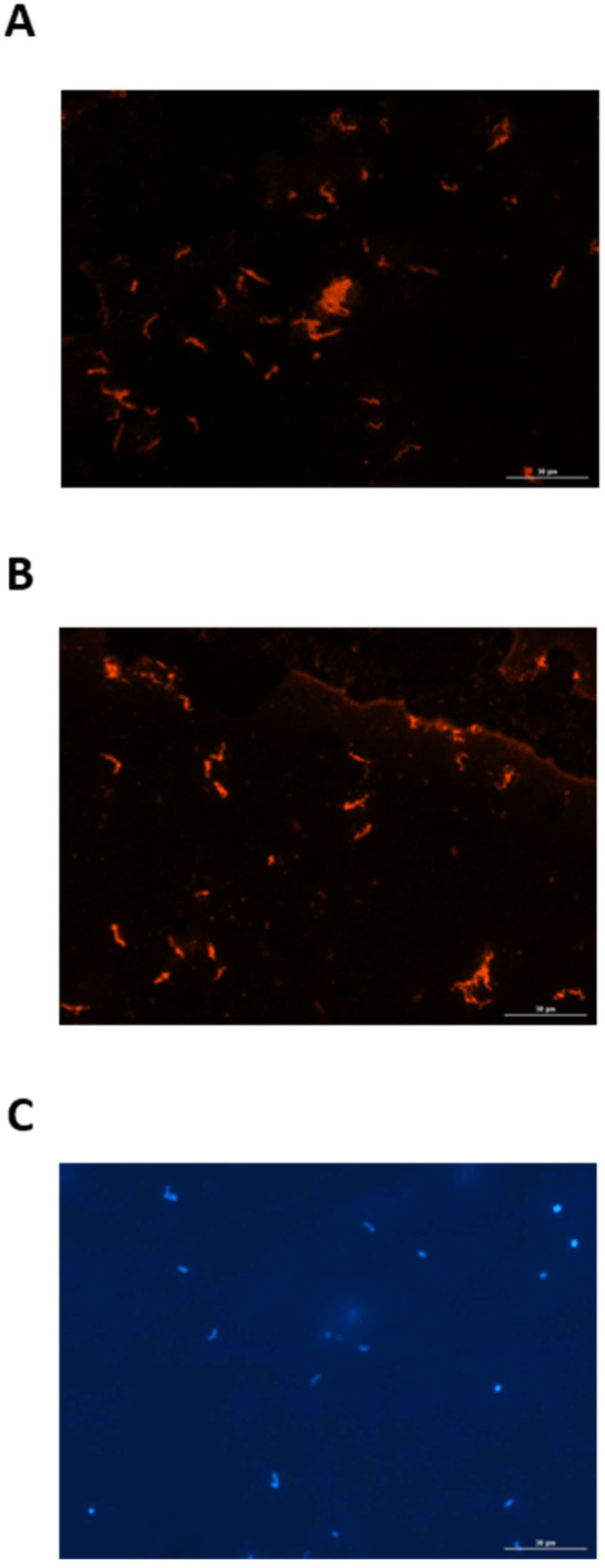
Representative fluorescent antibody test demonstrating that urinary-derived leptospires are reactive with (**A**) serum and (**B**) urine from experimentally infected rats. Urinary-derived leptospires are not reactive when probed directly with anti-IgG. The presence of leptospires in all samples tested was confirmed by DAPI (**C**). Original magnification ×400.

Since urine from experimentally infected rats contained IgG specific for leptospires, a FAT was used to determine whether leptospires excreted in urine from infected rats were bound to the urinary IgG. Urinary-derived leptospires were collected from experimentally infected rats at PIW4 (*n* = 8) and PIW8 (*n* = 8), and assessed for the presence of bound rat IgG by probing directly with anti-rat IgG, compared to control samples which were first reacted with serum or urine from infected rats. Controls assays readily detected urinary-derived leptospires by FAT when probed with serum from infected rats followed by detection with anti-rat IgG, as seen in Figure 3A. Similarly, urinary-derived leptospires were also readily detected when probed with urine from infected rats followed by detection with anti-rat IgG, Figure 3B. However, in no case were urinary-derived leptospires detected when probed directly with anti-rat IgG, indicating that leptospires are not bound to urinary IgG during active excretion. All FAT assays included a DAPI control, as presented in Figure 3C.

**Figure 4:**
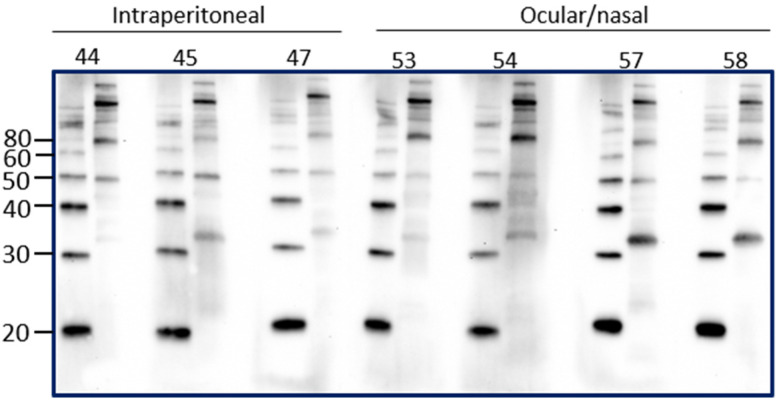
Detection of urinary IgG specific for leptospires in urine from inbred female rats experimentally infected using the intraperitoneal route compared to the ocular/nasal route. Representative immunoblots of leptospires with urine collected at PIW4. Molecular mass markers (M) are indicated in kDa and are shown in the left lane of each immunoblot compared to antigen of leptospires in the right lane.

Intraperitoneal inoculation of laboratory animal models of leptospirosis provides a reproducible route of inoculation with a known dose that can allow for a predictable and reproducible course of infection and shedding of leptospires. However, the intraperitoneal route of inoculation is not reflective of natural exposure. In order to determine if the presence of urinary IgG in experimentally infected rats reflected the route of inoculation, urinary IgG was assessed in rats that were inoculated by an ocular/nasal route compared to those infected by the intraperitoneal route. Results demonstrate that IgG specific for leptospires is present in urine at PIW4 in rats inoculated by the ocular/nasal route, as seen in Figure 4.

**Figure 5:**
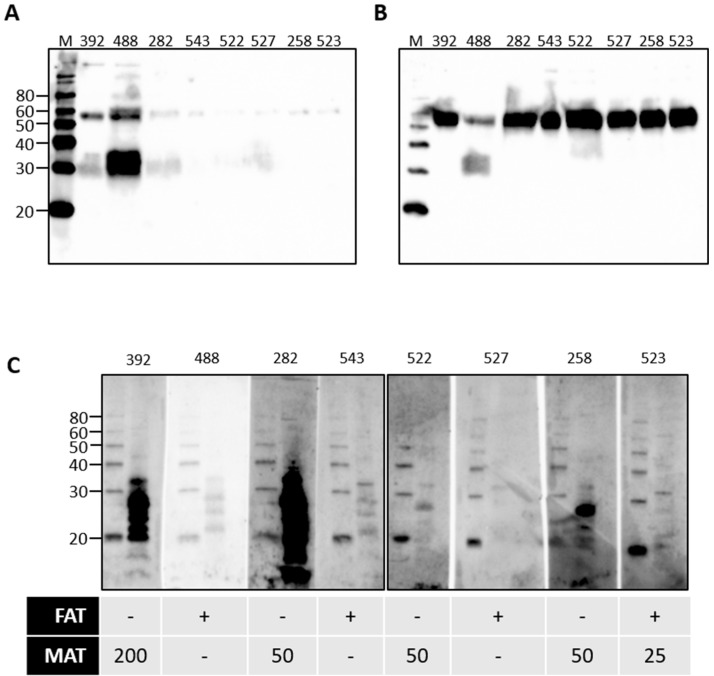
Detection of urinary IgG (**A**) or urinary IgA (**B**) in the urine of cattle that were naturally exposed to leptospires. Urinary IgA specific for leptospires from naturally infected cattle was demonstrated by immunoblotting of leptospires (**C**). The diagnostic status of each animal (numbered at the top of each gel) is provided for the fluorescent antibody test (FAT-positive [+] or -negative [-]) and the microscopic agglutination test (MAT) at 1:25 or above. Molecular mass markers (M) are indicated in kDa.

Rats are a reservoir host for *L*. *interrogans* serovar Copenhageni and cattle are a reservoir host for *L*. *borgpetersenii* serovar Hardjo. Given our observations for the presence of urinary immunoglobulin in experimentally infected rats, urine samples from cattle naturally exposed to *L*. *borgpetersenii* were assessed for the presence of immunoglobulin. Our previous work demonstrated that 20% of beef cattle were seropositive for leptospires, as determined by the microscopic agglutination test (MAT) [5]. In addition, 7.2% of beef cattle were actively excreting leptospires in urine, as determined by FAT and culture. FAT-positive cattle can be seronegative by MAT [4,5]. A representative subset of bovine urine samples from our previous work were assessed for the presence of urinary Ig specific for leptospires, as presented in Figure 5. Given the potential for urinary Ig to act as a diagnostic marker for the detection of cattle that are actively shedding leptospires in the absence of a seropositive MAT response, the subset comprised samples that were from cattle that were MAT-positive and FAT-negative, or from cattle that were FAT-positive and MAT-negative. In contrast to rats, IgA was readily detected in all bovine urine samples tested, shown in Figure 5B, compared to IgG, shown in Figure 5A. The specificity of bovine urinary IgA in cattle that were MAT- or FAT-positive for leptospires was confirmed by immunoblotting, as seen in Figure 5C. Those cattle which are MAT-positive but FAT-negative appear to have higher levels of urinary IgA specific for antigens of leptospires compared to those cattle which are MAT-negative but FAT-positive.

## 4. Discussion

Aside from being a significant reservoir host and source of infectious *Leptospira* that may cause acute leptospirosis in human and domestic animal populations, rats provide a convenient laboratory model to elucidate the unique biological equilibrium between pathogenic *Leptospira* species and their respective reservoir host of infection [24]. Urine from experimentally infected rats provides the ability to collect urinary derived leptospires to understand how leptospires adapt to, and persist in, their host during infection as well as the ability to characterize the protein composition of urine to determine the host’s pathophysiological response to infection [8,17]. Experimentally infected rats have previously been shown to excrete leptospires despite a robust humoral and cellular immune response. In this study, we demonstrate that urine from experimentally infected rats also contains immunoglobulin specific for protein antigens of *Leptospira*.

Early work on acute leptospirosis demonstrated the presence of agglutinating antibodies in urine of patients [25,26]. Agglutination titers were also identified in the urine of carrier mice; the antibodies present were active against in vitro cultured leptospires but inactive against renal derived leptospires [27]. These results are supported by more recent work which has demonstrated that urinary derived leptospires modify expression of antigens, as well as their respective post translational modifications during persistent renal infection compared to in vitro growth [8,14,15] and support the finding that urinary IgG was not bound to leptospires during active excretion. The failure to detect urinary IgG bound to excreted leptospires suggest that they are either not being exposed to antibodies, or are exposed at such a low level, or to poorly avid antibodies, that any antibody, if bound, is not detected by the method employed. Alternatively, it is reflective of the induced diuresis and that leptospires displaced from the renal tubule are not exposed to an antibody unless or until they are retained in the bladder. The presence of urinary IgG was detected regardless of whether rats were inoculated via the intraperitoneal route, or the ocular/nasal route of infection.

Domestic animals also act as reservoir hosts of leptospirosis, including cattle which act as a reservoir host for different species and serovars to that of rats. In order to determine if urinary excretion of Ig specific for leptospires was a universal feature of reservoir hosts of infection, we assessed urine from cattle that were naturally exposed to, and infected with, *L*. *borgpetersenii* serovar Hardjo. A representative subset of bovine urine samples determined that IgA was readily detected in all urine samples tested but did not differentiate those animals with active shedding from previous exposure. Whether urinary IgA from cattle actively shedding leptospires can detect and differentiate specific protein antigens of *Leptospira* compared to urinary IgA in vaccinated or exposed cattle remains to be determined. Cattle infected with serovar Hardjo can excrete leptospires for more than one year [28]. In experimentally infected cattle, cessation of leptospiruria was associated with a sharp increase in urinary anti-*Leptospira* IgG and IgA antibody levels [29]. Those mechanisms that facilitate such a host response and clearance of infection remain to be determined.

## Data Availability

Data is contained within the article.
